# Aptamer-Based High-Throughput Screening Model for Efficient Selection and Evaluation of Natural Ingredients against SGIV Infection

**DOI:** 10.3390/v14061242

**Published:** 2022-06-08

**Authors:** Hongling Wei, Zhongbao Guo, Yu Long, Mingzhu Liu, Jun Xiao, Lin Huang, Qing Yu, Pengfei Li

**Affiliations:** 1 Guangxi Engineering Research Center for Fishery Major Diseases Control and Efficient Healthy Breeding Industrial Technology (GERCFT), Guangxi Key Laboratory of Aquatic Biotechnology and Modern Ecological Aquaculture, Guangxi Academy of Sciences, Nanning 530007, China; weihongling8@163.com (H.W.); liumz1988@126.com (M.L.); hlin556@163.com (L.H.); 2 Guangxi Key Laboratory of Aquatic Genetic Breeding and Healthy Breeding, Guangxi Academy of Fishery Science, Nanning 530000, China; guozhongbaono1@163.com (Z.G.); dreamshaw@foxmail.com (J.X.); 3 Department of Biochemistry and Molecular Biology, Wuzhou Medical College, Wuzhou 543000, China; xiaolin9902@163.com

**Keywords:** Singapore grouper iridovirus, aptamer, high-throughput screening, medicinal plants components, antiviral effect

## Abstract

Singapore grouper iridovirus (SGIV) causes high economic losses in mariculture. Effective drugs for managing SGIV infection are urgently required. Medicinal plant resources are rich in China. Medicinal plants have a long history and significant curative effects in the treatment of many diseases. Reverse-transcription quantitative real-time PCR is the most commonly used method for detecting virus infection and assessing antiviral efficacy with high accuracy. However, their applications are limited due to high reagent costs and complex time-consuming operations. Aptamers have been applied in some biosensors to achieve the accurate detection of pathogens or diseases through signal amplification. This study aimed to establish an aptamer-based high-throughput screening (AHTS) model for the efficient selection and evaluation of medicinal plants components against SGIV infection. Q2-AHTS is an expeditious, rapid method for selecting medicinal plant drugs against SGIV, which was characterized as being dram, high-speed, sensitive, and accurate. AHTS strategy reduced work intensity and experimental costs and shortened the whole screening cycle for effective ingredients. AHTS should be suitable for the rapid selection of effective components against other viruses, thus further promoting the development of high-throughput screening technology.

## 1. Introduction

Grouper (*Epinephelus* spp.) is one of the most valuable marine aquaculture fish in the coasts of southern China and Southeast Asian countries. Grouper production reached 192,045 tons in 2020 in China. As a result of grouper’s richness, rapid growth, and high market value, the species is significant in both fisheries and aquaculture in Southern China [1]. In recent years, the rapidly expanding scale of grouper aquaculture and the abuse of antibiotics have increased the incidence of frequent diseases, which seriously affects the healthy and sustainable development of grouper aquaculture [2]. Singapore grouper iridovirus (SGIV) is one of the most serious viral pathogens in grouper aquaculture. Typically, SGIV infection causes blackening of the body, weakened swimming ability, and even lethargy, resulting in high mortality and serious economic losses [3,4].

The traditional method of preventing aquatic diseases is relying on chemical drugs, but it can easily cause drug resistance and drug residues are left in the body. Therefore, medicinal plants have been widely applied because of their non-toxicity, non-tolerance, and low cost. A growing number of studies have reported that the resources of medicinal plants are rich in China and have been used for the treatment of numerous diseases for thousands of years [5]. Medicinal plants show obvious advantages. For example, they have lots of active ingredients with antibacterial and antiviral activities [6,7,8]. They could improve the anti-stress ability [9], prevent drug resistance of pathogens and are biodegradable and biocompatible [10]. It is worth noting that a variety of medicinal plant extracts have been widely used due to their significant antibacterial and antiviral effects [11,12,13]. For example, Ma et al. [14] found that 27 Chinese medicinal plants exhibited potent activity against respiratory syncytial virus. Wang et al. [15] found that all total flavonoids extracted from the floral buds of *Lonicera japonica* Thunb. demonstrated potent activity against influenza virus H9N2. However, the chemical composition of natural medicinal plants is complex, so it is important to develop methods for evaluating the effective ingredients from natural medicinal plants. Nowadays, the most frequently used means of evaluating the antiviral activities of natural medicinal plants is reverse-transcription quantitative real-time PCR (RT-qPCR). RT-qPCR is sensitive, but high in reagent costs, and complex and time-consuming procedures limit their application. Therefore, it is important to develop a high-throughput rapid screening technology for antiviral drugs to improve the rate of discovery of effective components, which could reduce economic losses [16].

Aptamers are synthetic oligonucleotides (generally 20–100 nucleotides), such as ssDNA or RNA, or protein ligands, which are selected from a large combinatorial library of nucleic acids by RT-PCR and PCR in vitro through system evolution of ligands based on exponential enrichment technology (SELEX). This technique was first reported in 1990 [17]. Aptamers usually have a characteristic three-dimensional structure and are characterized by complex structural features, such as stem-loops, hairpins, and pseudoknots, and they are easily synthesized and have low toxicity, easy modification, strong stability, and specific recognition and binding of target substances [18,19], allowing them to be used as excellent molecular probes in biological applications [20]. Extensive literatures have reported that aptamers have been applied in multiple applications rather than antibodies, such as medical diagnostic, bio-sensing, and bio-imaging platforms. Antibodies are usually bound to proteins for diagnosis; however, aptamers can bind to a variety of targets, such as proteins [21], bacteria/pathogens [22], viruses [23], live cancer cells [24], and tissues [25]. Many studies have proved that aptamers can monitor the virus infection process [26,27,28,29]. Some aptamers have been used to establish aptamer-based screening models, including the aptamer-based fluorescent molecular probe assay [30].

In our previous study, we have successfully generated highly specific aptamers against SGIV-infected grouper spleen (GS) cells [19]. Some aptamers have the potential to form aptamer-based screening models. Hence, in this study, we developed an aptamer (Q2)-based high-throughput screening (Q2-AHTS) technique for efficient selection and evaluation of natural ingredients against SGIV infection, which is important for improving the prevention and control of aquatic animal pathogens.

## 2. Materials and Methods

### 2.1. Cell Lines, Virus, and Reagents

GS cells were cultured in Leibovitz’s L15 medium (Gibco, Grand Island, NY, USA) containing 10% fetal bovine serum (Thermo Fisher Scientific, Waltham, MA, USA) at 28 °C. SGIV was isolated from the hybrid grouper (*Epinephelus fuscoguttatus*♀ × *Epinephelus lanceolatus*♂) [2]. The virus strain SGIV was propagated in GS cells and kept at −80 °C in the laboratory. Medicinal plants and their ingredients (Table S1) were purchased from Nanning Yixin Pharmacy, Guangxi (Guangxi, China) and Chengdu Herb purify Co., Ltd. (Chengdu, China). Hoechst 33342 (C_27_H_28_N_6_O·3HCl·3H_2_O) was purchased from Beyotime Biotech company (Shanghai, China).

### 2.2. Aptamer Q2 to Monitor SGIV Infection

An aptamer Q2 is a synthetic oligonucleotide selected by SELEX technology. It recognizes grouper-iridovirus-infected cells with high specificity, no toxicity, and low immunogenicity [19]. Its sequence (95 nucleotides) was 5′-GACGCTTACTCAGGTGTGACTCGTATGTCCATGGCCGCATATTGGGAAGGTTGGTTTGGACTATGTGGAAGTTCGAAGGACGCAGATGAAGTCTC-3′. The 5′ position of Q2 was labeled with 6-carboxy-fluorescein (FAM) and synthesized by Sangon Biotech (Shanghai, China). It was applied to identify SGIV infection by fluorescence analysis (495/535 nm). GS cells (1.2 × 10^5^) were seeded into 96-well plates (Shengyou Biotechnology, Hangzhou, Zhejiang, China), incubated at 28 °C for 18 h, and infected with different multiplicity of infection (MOI) of SGIV and incubated for 48 h post-infection (hpi). On the other hand, GS cells were cultured in 96-well plates and infected with SGIV at an MOI of 0.5 for 6, 12, 24, and 48 h. FAM-labeled aptamer Q2 (FAM-Q2)(1000 nM) was heated at 95 °C for 5 min and cooled by ice for 5 min for denaturation. Finally, the cells were incubated with aptamer Q2 at 4 °C for 30 min, and cells were washed three times with PBS (phosphate-buffered saline, 10 mM Na_2_HPO_4_·12 H_2_O, 2 mM KH_2_PO_4_, 137 mM NaCl, 1‰ NaN_3_). Cells were collected for fluorescence analysis at 495/535 nm by Infinite M200 Pro microplate reader instruments (Tecan, Switzerland, Europe).

### 2.3. Confocal Fluorescence Imaging Analysis

For live cell fluorescent imaging, GS cells (1 × 10^5^) were cultured in 35 mm glass-bottom dishes (Cellvis, Hangzhou, Zhejiang, China) and infected with SGIV (MOI = 0.5) for 0, 6, 12, 24, and 48 h. The SGIV-infected cells were incubated with aptamer Q2 (1000 nM) at 4 °C for 30 min. Hoechst 33,342 (200 uL) was added to the cells for 5 min, and cells were carefully washed three times with PBS. Serum-free phenol-red-free medium (150 uL) was added to cells, and fluorescence was detected by laser scanning confocal microscopy (LSCM; Nikon, Tokyo, Japan). SGIV-infected GS cells at 0 hpi were used as the control.

### 2.4. Gene Expression Detection by RT-qPCR

GS cells (9.6 × 10^5^) were seeded in 12-well plates (Corning, New York, NY, USA) and cultured for 18 h. Cells without treatment served as control group 1, cells incubated with only SGIV served as control group 2, and medicinal plants components co-incubated with SGIV served as the experimental group. After treatments, total RNAs were extracted from cells using TRIzol Reagent (ComWin Biotech Co., Ltd., Beijing, China). The RevertAid First Strand cDNA Synthesis Kit (ComWin Biotech Co., Ltd., Beijing, China) was used to synthesize cDNA. The cDNAs were amplified by RT-qPCR using SYBR Green PCR Master Mix (Toyobo, Tokyo, Japan). SGIV infection was identified by detecting SGIV major capsid protein (*MCP*) transcripts, and the β-actin gene was used as the internal control [31]. The data were calculated using the 2^−ΔΔCT^ method [32]. The genes and primers used for quantitative RT-qPCR are listed in Table 1.

### 2.5. Percentage Inhibition of SGIV Infection by Q2-AHTS or RT-qPCR

The percentage inhibition of SGIV infection by medicinal plants’ components were assessed and calculated using the following formula: Percentage inhibition = 1 − [(x − b)/(a − b)] × 100%. In this formula, x represents the results for each group treated with SGIV and medicinal plants components, the results for cells treated with SGIV alone, and b the results of the normal GS cells [31].

### 2.6. Statistical Analysis

The average value of three independent experiments was calculated. Intergroup differences were compared using a one-way analysis of variance with SPSS 21.0 (IBM, Armonk, NY, USA). The results of comparisons with *p* < 0.05 were considered to indicate statistically significant differences.

## 3. Results

### 3.1. Flow Chart of Q2-AHTS and RT-qPCR Screening Technology

**Figure 1:**
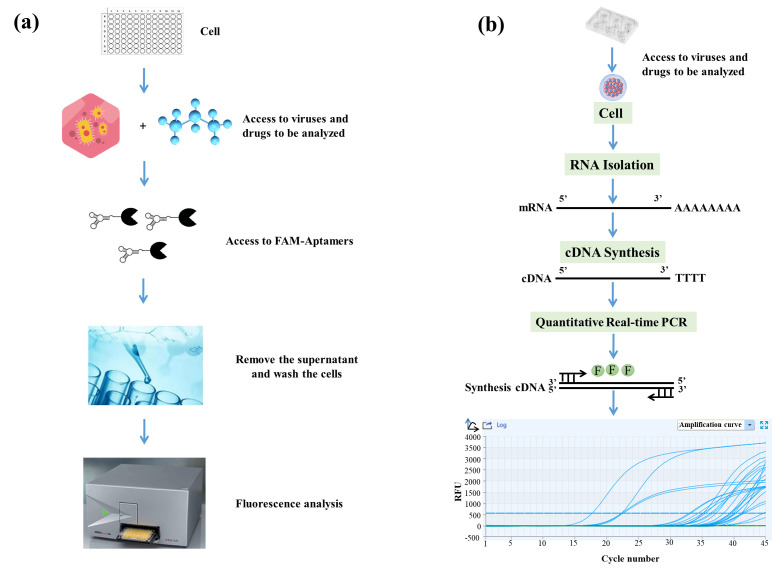
Flow chart of Q2-AHTS and RT-qPCR technology. (**a**) Flow chart of AHTS technology. (**b**) Flow chart of qPCR technology.

At present, the screening methods for SGIV mainly include microscopic observation, PCR, and flow cytometry methods. Phan et al. [33] established a high-throughput screening (HTS) model, which had dram, high speed, sensitivity, and accuracy. In a previous study, we found that aptamer Q2 could detect SGIV infection [19,31]. Hence, Q2-AHTS and RT-qPCR were used to detect SGIV infection and evaluate antiviral activity. Q2-AHTS required < 2 h and required only incubation, washing, and fluorescence measurement (Figure 1a). RT-qPCR required > 12 h from RNA extraction to results detection and analysis (Figure 1b).

### 3.2. Fluorescent-Labeled Aptamer Probe for Specific Detection of SGIV Infection

**Figure 2:**
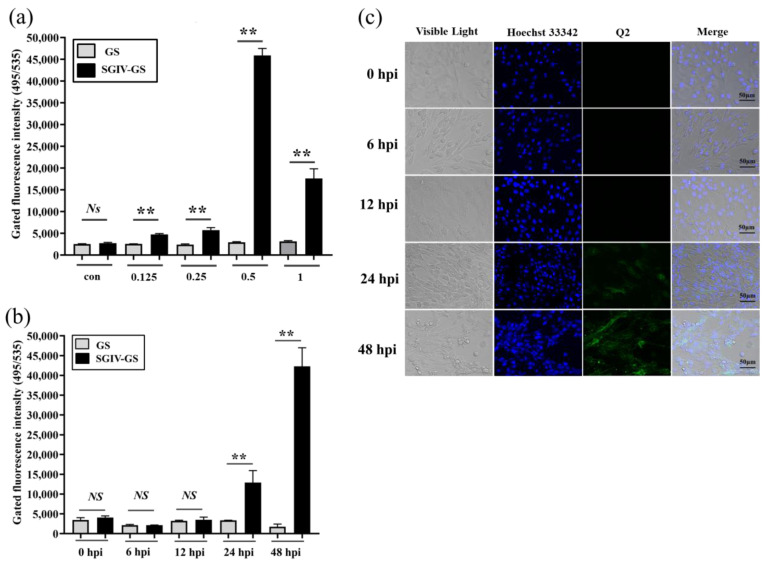
Monitoring SGIV infection by FAM-Q2. (**a**) Monitoring different MOI of SGIV infection by FAM-Q2. (**b**) Monitoring SGIV (MOI = 0.5) with different infection time by FAM-Q2. Fluorescence on SGIV-infected cells could be detected at 24 hpi, and the fluorescence increased over time. Results are presented as mean ± SD of three independent experiments (** *p* < 0.01). (**c**) LSCM showed the specific binding of FAM-Q2 to SGIV-infected cells but not to normal GS cells.

Virus infection usually causes changes in cell surface structures, and targeting these changes plays an important role in the development of new diagnostic and therapeutic methods [34,35]. FAM-Q2 was applied for the specific detection of SGIV infection. Compared with the control group of normal GS cells without SGIV infection, the fluorescent signal increased with the SGIV titer, which peaked in cells infected with SGIV at MOI of 0.5 (Figure 2a). Therefore, SGIV (MOI = 0.5) was selected for viral infection in subsequent experiments. We also explored how the fluorescent signal changed with time-dependent increase in SGIV infection. The binding of aptamer Q2 to SGIV-infected cells was significantly increased at 24 and 48 hpi (Figure 2b), which was consistent with confocal observation (Figure 2c). These results suggest that Q2 could monitor SGIV infection with specificity.

### 3.3. RT-qPCR for Specific Detection Analysis of SGIV Infection

**Figure 3:**
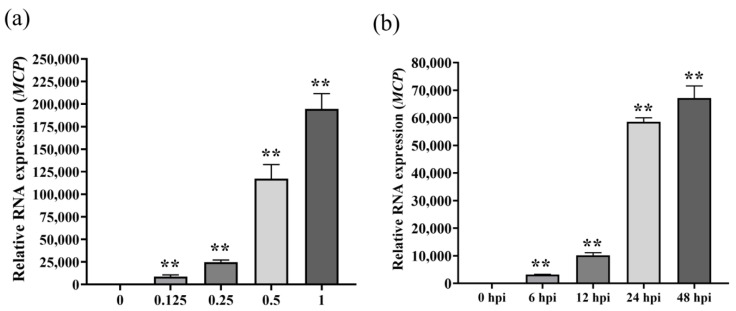
Monitoring SGIV infection by RT-qPCR. (**a**) RT-qPCR **detected** SGIV (MOI = 0.125) infection as early as 6 hpi. (**b**) RT-qPCR **detect** SGIV (MOI = 0.5) infection at 48hpi. Results are presented as mean ± SD of three independent experiments (** *p* < 0.01).

RT-qPCR was used to detect the expression of SGIV infection with different titers or infection times. Compared with normal GS cells without SGIV infection in the control group, the levels of viral *MCP* gene expression increased and showed significant differences with the increase in SGIV infection (Figure 3a). Viral *MCP* gene expression increased and showed a significant difference with the increased time of SGIV infection (Figure 3b). These results suggest that RT-qPCR could monitor SGIV infection.

### 3.4. Antiviral Activity Analysis of Medicinal Plants Components with Q2-AHTS

**Figure 4:**
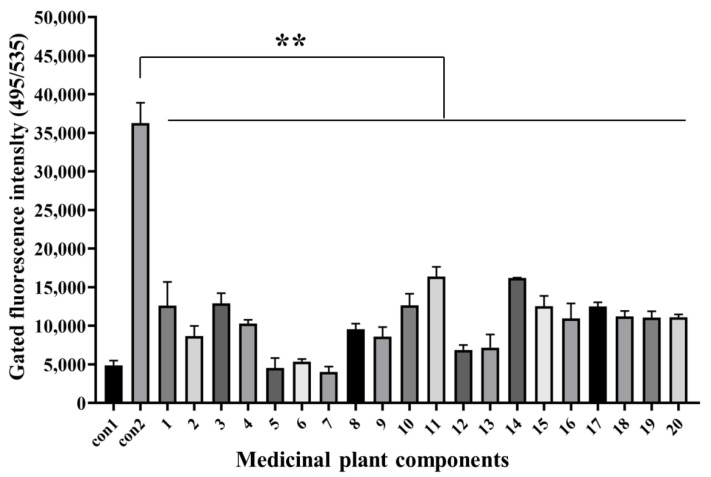
Antiviral analysis of 20 medical plant components against SGIV infection. AHTS results showed that fluorescence of target cells incubated with both SGIV and all **components (Nos. 1–20)** decreased obviously. Results are presented as mean ± SD of three independent experiments (** *p* < 0.01).

Twenty natural components (>98% purity) were isolated from different medicinal plants (Table S1), whose safe working concentrations were verified in the early stage of this study. GS cells incubated with components at safe working concentrations and infected with SGIV (MOI = 0.5) were the test group, normal GS cells without SGIV infection served as the control group1 (con1), and cells infected with SGIV alone served as the control group2 (con2). Compared with the control groups, the fluorescent signal of the test group (Nos. 1–20) decreased significantly, which indicated that these components had antiviral activity against SGIV infection (Figure 4).

### 3.5. Antiviral Activity Analysis of Medicinal Plants Components with RT-qPCR

**Figure 5:**
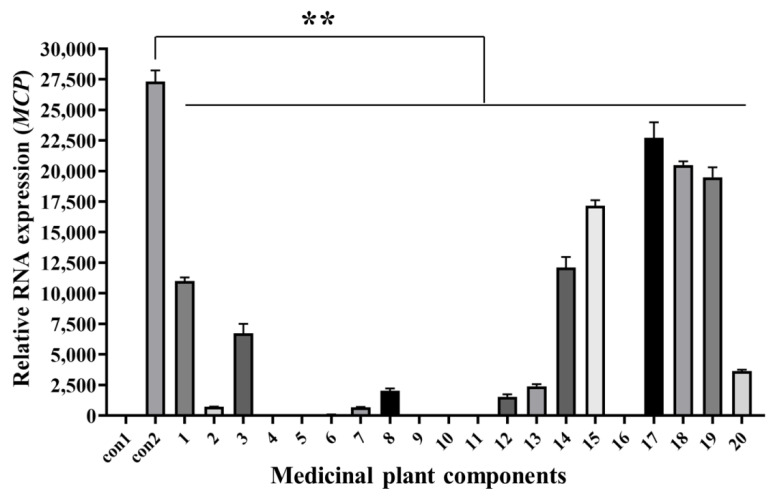
Antiviral analysis of 20 medicinal plant components against SGIV infection. RT-qPCR showed that the expression of *MCP* gene decreased after treatment with components 1–20. Results are presented as mean ± SD of three independent experiments (** *p* < 0.01).

The cells and culture supernatants at 48 hpi were collected for RT-qPCR analysis. *MCP* gene expression of cells incubated with both SGIV and all components (Nos. 1–20) decreased significantly compared with that in the control groups (Figure 5). This indicates that these components had antiviral activities against SGIV infection. RT-qPCR results were consistent with Q2-AHTS results.

### 3.6. Comparison of Inhibition Rates between Q2-AHTS and RT-qPCR

**Figure 6:**
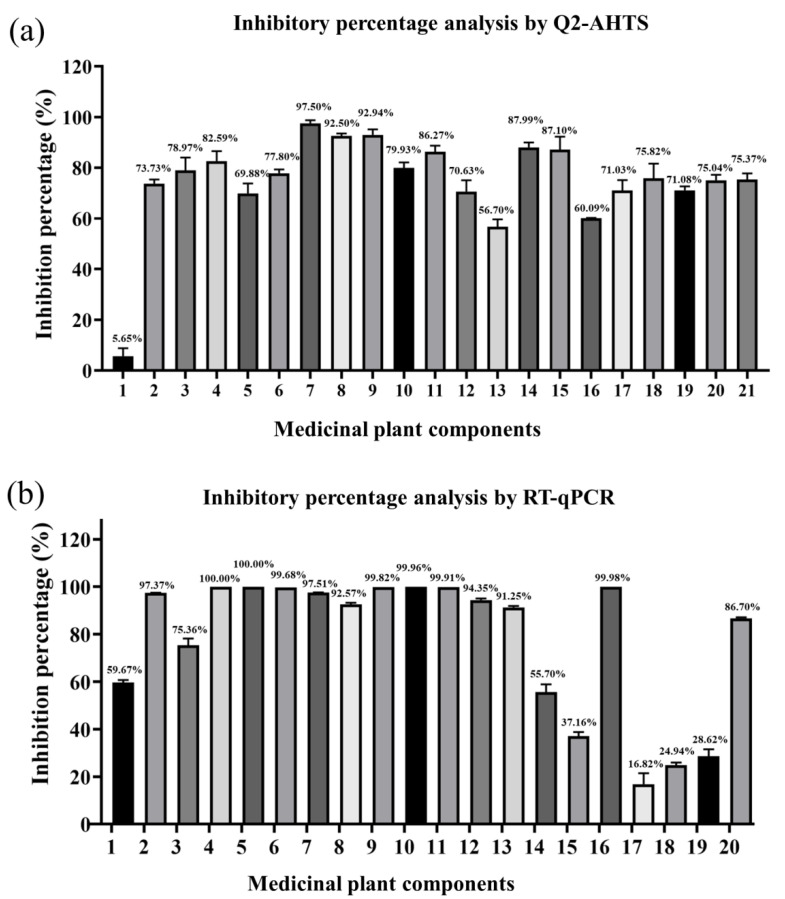
Inhibitory percentage of medicinal plant components against SGIV infection analyzed by Q2-AHTS (**a**) and RT-qPCR (**b**).

The percentage inhibition of medicinal plant components against SGIV infection was evaluated by Q2-AHTS or RT-qPCR. Q2-AHTS showed that the 20 components exhibited significant inhibitory activity > 50% (Figure 6a). RT-qPCR showed that components 1–14, 16, and 20 exhibited > 50% inhibitory activity, whereas 15 and 17–19 exhibited < 50% inhibitory activity (Figure 6b). Overall, the results of Q2-AHTS showed that the trend for most medicinal plant components’ (Nos. 1–14, 16, and 20) anti-SGIV effects were consistent with RT-qPCR screening techniques. Nevertheless, some components (Nos. 15 and 17–19) exhibited different trends.

## 4. Discussion

Rapid development of grouper aquaculture has resulted in large-scale outbreaks of diseases. SGIV infection had a particularly serious effect on grouper aquaculture [35]. Effective drugs against SGIV infection are urgently needed. Medicinal plants have a long history and have been widely used in the treatment of various diseases [36]. Some methods for screening antiviral drugs have been developed based on molecular biology, immunology, pathological tissue observation and transmission electron microscopy. For example, Li et al. [37] observed the antiviral effect of Lianhuaqingwen on SARS-CoV-2 using electron microscopy. Liu et al. [8] found *Lonicera japonica* Thunb. components had anti-SGIV activities in vitro and in vivo by RT-qPCR. Takashita et al. [38] used ELISA to screen antiviral drugs against SARS-CoV-2 variants in vitro. Although these methods have high sensitivity and accuracy, there are some disadvantages, such as cumbersome operation, expensive instruments, long detection time, and harsh reagent storage conditions. Additionally, these methods do not meet the needs of rapid screening of antiviral drugs. Therefore, less time-consuming, less expensive, and rapid screening antiviral drug methods for SGIV are urgently needed. Aptamers are new detection probes with many advantages, such as stability, easy synthesis, high affinity, and specificity. They have been widely applied in many important areas, including development of potential biomarkers, pathogen detection, and cell-specific drugs delivery [30]. It has been reported that AHTS [39] could monitor pathogens and evaluate effective agents with antiviral activities. Therefore, we developed Q2-AHTS for anti-SGIV drugs.

We first explored the specificity and sensitivity of Q2-AHTS for the detection of SGIV infection. The fluorescence results showed that the binding of aptamer Q2 to SGIV-infected cells (MOI = 0.5) was significantly increased at the 24 and 48 hpi, which was consistent with confocal observation. Aptamer Q2 is less sensitive to SGIV than aptamer LYGV1 [40], which may be because the dissociation equilibrium constant (Kd) of aptamer LYGV1 (Kd = 32.55 nmol/L) is higher than that of aptamer Q2 (Kd = 12.09 nmol/L). Second, we explored the specificity and sensitivity of detecting SGIV infection by RT-qPCR and Q2-AHTS. RT-qPCR showed that viral *MCP* gene expression increased and showed a significant difference with the increasing virus titer and time of SGIV infection. The results were consistent with a previous published study [40]. By comparing the specificity and sensitivity of RT-qPCR and Q2-AHTS for identifying SGIV infection, we found that two screening methods had the same trends at 24 and 48 hpi of SGIV infection and had different trends at 6 and 12 hpi, indicating that the detection accuracy of AHTS was not comparable to that of RT-qPCR. Therefore, we believe that the Q2-AHTS screening technique needs to be optimized in future studies.

Traditional antibiotics and other chemical drugs can cause serious public safety problems. In comparison, medicinal plants have been used to treat numerous diseases for thousands of years and are being increasingly used in aquaculture because of their simple application, cost-effectiveness, and high efficiency [36,41]. In this study, we used Q2-AHTS and RT-qPCR screening technology to screen antiviral activity of 20 medicinal plants. Both of these methods proved that 20 natural component isolated from different medicinal plants had antiviral activities against SGIV infection. The anti-SGIV effects of components 1–14, 16, and 20 by Q2-AHTS were consistent with those measured by RT-qPCR, which was consistent with previous studies [40]. Nevertheless, the antiviral effects of components 15 and 17–19 differed when measured by Q2-AHTS and RT-qPCR. This indicates that the detection accuracy of AHTS is less than that of RT-qPCR, and the aptamer used in AHTS requires more structural optimization and specific affinity enhancement to achieve the aim of improving detection accuracy of AHTS.

In conclusion, RT-qPCR screening is complex, expensive and time-consuming, which makes it hard to perform rapid analysis of numerous active medicinal plant components against virus infection. In contrast, Q2-AHTS screening could reduce the work load and experimental costs and shorten the screening time of effective components. Therefore, the application prospects of AHTS technology are broad in the field of high-throughput rapid screening of antiviral drugs, which is important for improving the prevention and control of aquatic diseases.

## Figures and Tables

**Table 1:** Primers for detecting SGIV infection by RT-qPCR.

Primer	Sequences
qMCP-F	5′-GCACGCTTCTCTCACCTTCA-3′
qMCP-R	5′-AACGGCAACGGGAGCACTA-3′
β-actin-F	5′-TACGAGCTGCCTGACGGACA-3
β-actin-R	5′-GGCTGTGATCTCCTTCTGCA-3′

## Data Availability

The data that support the findings of this study are available from the corresponding author upon reasonable request.

## Supplementary Materials

The following supporting information can be downloaded at: https://www.mdpi.com/article/10.3390/v14061242/s1, Table S1: The properties of natural ingredients from medicinal plants.
