# Supplementary material for: Aptamer-Based High-Throughput Screening Model for Efficient Selection and Evaluation of Natural Ingredients against SGIV Infection
Source: Viruses. 2022 Jun 8;14(6):1242. doi: 10.3390/v14061242 (PMC9227401; doi:10.3390/v14061242)
Supplement: Supplementary file 1 [file viruses-14-01242-s001.zip › viruses-1713852-supplementary.pdf]

Table S1. The properties of natural ingredients from medicinal plants.

Number	Name	Origin	Safe concentrations
1.	*Green tea* aqueous extract	Green tea	≤ 100 μg/mL
2.	(-)-Epigallocatechin	Green tea	≤ 50 μg/mL
3.	(-)-Epicatechin	Green tea	≤ 20 μg/mL
4.	Tea Polyphenol	Green tea	≤ 10 μg/mL
5.	(-)-Epigallocatechin gallate	Green tea	≤ 10 μg/mL
6.	(-)-Epicatechin gallate	Green tea	≤ 10 μg/mL
7.	*Illicium verum* aqueous extract	*Illicium verum* Hook. f.	≤ 500 μg/mL
8.	Shikimic acid	*Illicium verum* Hook. f.	≤ 250 μg/mL
9.	3,4-dihydroxybenzoate acid	*Illicium verum* Hook. f.	≤ 400 μg/mL
10.	quercetin	*Illicium verum* Hook. f.	≤ 50 μg/mL
11.	*Areca catechu* aqueous extract	*Areca catechu L.*	≤ 31.25 μg/mL
12.	Arecoline hydrobromide	*Areca catechu L.*	≤ 125 μg/mL
13.	*Agrimonia pilosa* aqueous extract	*Agrimonia pilosa*	≤ 156.25 μg/mL
14.	*Mulberry Leaves* aqueous extract	*Mulberry Leaves*	≤ 500 μg/mL
15.	Isoquercitrin	*Mulberry Leaves*	≤ 1000 μg/mL
16.	Morin	*Mulberry Leaves*	≤ 100 μg/mL
17.	β-sitosterol	*Mulberry Leaves*	≤ 12.5 μg/mL
18.	Trihydroxyethylrutin	*Mulberry Leaves*	≤ 125 μg/mL
19.	*Magnoliae Officinalis Cortex aqueous* extract	*Magnoliae Officinalis Cortex*	≤ 500 μg/mL
20.	Apigenin	*Viola yedoensis*	≤ 1.25 μg/mL
